# Drone-based sputum transport for TB diagnosis in remote communities

**DOI:** 10.5588/ijtldopen.25.0484

**Published:** 2026-02-11

**Authors:** B. Kamble, V. Ganji, K. Nigam, S. Aggarwal, G. Potukuchi, R. Kundapur, M. Panda, N. Agarwal, V. Bhatia

**Affiliations:** 1 Departments of Community and Family Medicine, All India Institute of Medical Sciences (AIIMS), Bibinagar, India;; 2 Department of Physiology, All India Institute of Medical Sciences (AIIMS), Bibinagar, India;; 3 ICMR i-DRONE, Division of Descriptive Research, Indian Council of Medical Research-Hq, New Delhi, India;; 4 Department of CFM, AIIMS, Bhubaneswar, Odisha;; 5 All India Institute of Medical Sciences, Bibinagar, India.

**Keywords:** tuberculosis, Yadadri-Bhuvanagiri, India, unmanned aerial vehicle, turnaround time, rural health, health technology

## Abstract

**BACKGROUND:**

TB remains a major public health concern in India where diagnostic delays persist. This study assessed the impact of drone-based sputum transport on turnaround time (TAT) and access to TB care in Telangana’s Yadadri-Bhuvanagiri district.

**METHODS:**

A quasi-experimental mixed-methods study was conducted under the Indian Council of Medical Research’s i-DRONE initiative. Intervention included pre-drone phase (ground transport of samples) and drone phase (drone-based transport of samples). Outcomes: TAT, patient and diagnostic delay, and out-of-pocket expenditure (OOPE). Quantitative data were analysed using Jamovi and MS Excel; qualitative insights were gathered from open-ended remarks and observations made during the field.

**RESULTS:**

A total of 840 individuals (206 in the pre-drone phase and 634 in the drone phase) were enrolled. The median TAT reduced from 15 days (interquartile range [IQR]: 10–20) in the pre-drone phase to 5 days (IQR: 2–9) in the drone phase, and the mean (standard deviation) TAT dropped from 16.6 days (18.1) to 6.9 days (8.3) (*P* < 0.001). The mean OOPE declined from INR 9,451 (∼USD 113.4) to INR 90.9 (∼USD 1.0). Patients reported travel difficulties, loss of daily wages, and stigma as reasons for delaying care.

**CONCLUSION:**

Significant reduction in the TAT and improved access to TB diagnosis in rural and remote Indian settings support the feasibility of scaling drone-based logistics within national TB elimination efforts.

TB continues to be one of the most pressing infectious diseases worldwide, with India carrying one of the highest reported burdens.^1^ Although molecular diagnostics such as GeneXpert and Truenat have transformed testing capacity, the timely transport of sputum specimens remains a critical logistical weakness especially in rural and hilly regions, poor road infrastructure, unpredictable weather, and long distances to laboratories further impede reliable and timely transport.^2–4^ Delays not only deter treatment adherence and reduce follow-up rates but also elevate the risk of community spread.^5–7^ Given the severity of the TB epidemic across the world, innovative solutions are needed to ensure access for patients to quality diagnostic services. Unmanned aerial vehicles (UAVs), commonly referred to as drones, are increasingly being considered for public health logistics, and their use has demonstrated effectiveness in the delivery of essential medical commodities, including vaccines, blood products, medicines, and diagnostic samples.^8^ For instance, UAV deployment for early infant HIV diagnosis in Malawi significantly reduced diagnostic timelines.^9^ Similar feasibility studies in Papua New Guinea and northeast India have documented the practicality of drone-facilitated sputum transport, demonstrating improvements in speed, consistency, and operational feasibility in geographically challenging environments.^9^ In Ghana, at-scale aerial logistics for vaccine distribution was shown to be not only operationally feasible but also highly cost-effective, with incremental cost-effectiveness ratios as low as USD 41 per averted disability-adjusted life year from a societal perspective.^10^

Despite this growing evidence base, most prior studies have been limited in duration, restricted to pilot operations, or have focused on commodities other than TB sputum, leaving important gaps in understanding their impact on TB diagnostic pathways and comparing the drone-based sputum transport directly with conventional method, where individuals often spend an entire day journeying from remote villages to TB units (TUs) equipped with facilities. By addressing these transportation barriers, the present study aims to evaluate the effect of drone-based sputum transport on TB diagnosis turnaround time (TAT) in the Yadadri-Bhuvanagiri district of Telangana, India. By using drone technology, this study seeks to address critical barriers to TB diagnosis and enhance drone-based interventions for TB diagnostics, contributing to national and global TB elimination efforts.

## METHODS

This quasi-experimental pre–post study conducted in rural Telangana under the Indian Council of Medical Research’s (ICMR) i-DRONE initiative evaluated the impact of drone-based sputum transport on TAT and the out-of-pocket expenditure (OOPE) for TB diagnosis. The pre-drone phase (February–December 2023) relied on conventional ground-based travel, in which patients travelled from their villages to TUs. In the interventional drone phase (February–February 2025), sputum samples were transported by drones from peripheral health facilities, including primary health centres (PHCs) and subcentres, to the designated TUs. The study took place in the Yadadri-Bhuvanagiri district (population 770,833; Census 2011),^11^ which covers 3,253 km^2^ and 17 revenue mandals (administrative sub-district units within a district, each with a separate local governance structure). This district is characterised by hilly terrain, poor road connectivity, limited public transport, and a geographically dispersed health care network.

### Study site selection

**Figure 1.**
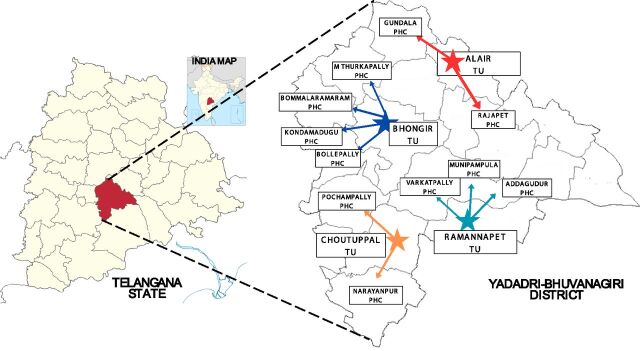
Administrative map of the Yadadri-Bhuvanagiri district of Telangana. The map illustrates the operational flow from the central command centre at AIIMS Bibinagar to the District TB Centre, 4 TB Units (TUs), and 11 peripheral primary health centres (PHCs) covered under the drone intervention.

Four TUs, and 11 PHCs, with their 60 subcentres, were purposively selected based on the TB burden, remoteness, and lack of TB diagnostic facilities (Figure 1). A total of 840 participants were included: 206 TB patients in the pre-drone phase and 634 presumptive TB patients in the drone phase.

### Implementation site

**Figure 2.**
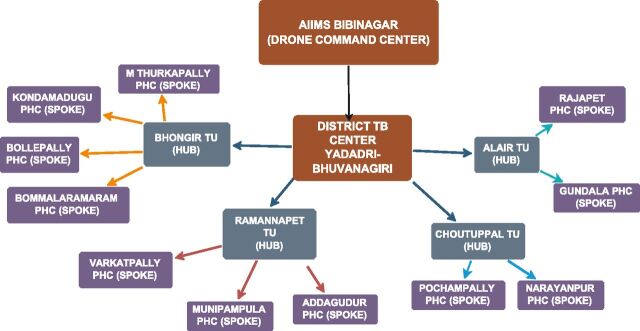
Schematic overview of the drone-enabled sputum transport network in Yadadri-Bhuvanagiri District, Telangana.

**Figure 3.**
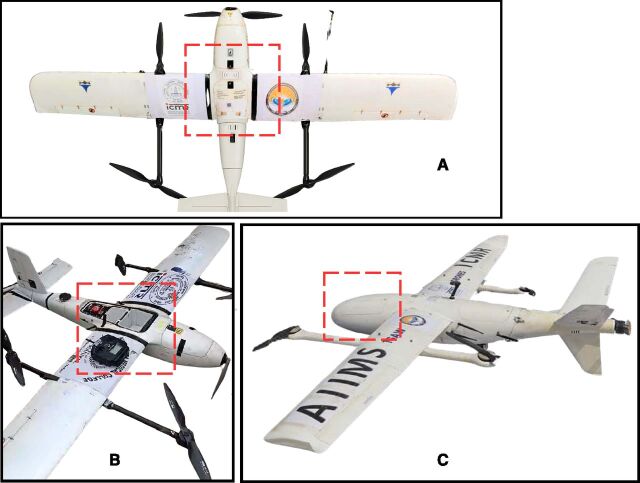
Images of **(A, B)** AD250 Drone and **(C)** AD350 drone used for sputum transport; the red box highlights the secure storage compartment with a protective lid.

A centralised command centre at AIIMS Bibinagar coordinated the drone operations (Figure 2). A licensed pilot managed drone deployment, live monitoring, and flight operations. Safe take-off and landing sites were identified at all sites (11 PHCs, 60 subcentres, and 4 TUs) and were selected for proximity to health facilities and operational feasibility under the Drone Rules, 2021.^12^ Two types of small-category vertical take-off and landing drones were used: i) AD250: (11.5 kg, capacity of 2 kg) and ii) AD350 (24 kg, capacity of 8 kg) (see Figure 3). Detailed flight routes were pre-programmed using the Key Markup Language (KML) file, considering terrain, population density, and no-fly zones.

### Development and execution of sortie plan

A hub-and-spoke model was established for drone-based sputum sample transport, with PHCs and their subcentres acting as spokes and TUs with Cartridge-Based Nucleic Acid Amplification Test (CBNAAT)/Truenat facility serving as diagnostic hubs (Figure 2). Drones were dispatched from the central command centre at AIIMS Bibinagar to peripheral PHCs and subcentres situated within villages. Sputum samples collected at these facilities were then transported by drone to the designated TUs as per pre-planned drone schedules. This system allowed drones to collect samples directly from the health facilities within villages and deliver them to TUs, thereby eliminating the need for patient travel beyond local facilities.

### Data collection

Data were collected using patient records and pre-validated questionnaires adapted from national TB frameworks, and tools were pretested and refined before deployment. We examined the TAT, the delay components: patient and diagnostic delay, and OOPE incurred by patients based on the WHO framework and definitions adopted from Boro et al.^13^ Operational definitions and OOPE category are given in Table 1.

**Table 1.** Operational definition of the variables used in this study.

Variable	Operational definition
Patient delay (days)	Time from onset of TB-related symptoms to first contact with the health care provider.
Diagnostic delay (days)	Time from first health care contact to confirmation of TB diagnosis.
Turnaround time (days)	The time taken from the first health care visit to the visit to the diagnostic facility.
Out-of-pocket expenditure (OOPE) (₹)	OOPE is calculated as direct medical costs (consultation, medication, diagnosis) + nonmedical costs (cost of travel for diagnosis and food) + indirect costs (travel for medication, wage loss of patient and attender).

### Patient and sample pathways, collection, and packaging

In the pre-drone phase, the patient pathway required the TB suspects travel 10–30 km by road, often with limited transport options, to TUs equipped with Cartridge-Based Nucleic Acid Amplification Test (CBNAAT)/Truenat facilities, as nearby PHCs and subcentres lacked diagnostic capacity. In the drone phase, the sample pathway replaced patient travel and patients submitted sputum at their nearest PHC or subcentre, usually within or near their village. Sputum samples were collected at PHCs/subcentres by trained health care workers following National TB Elimination Programme (NTEP) guidelines.^14^ Samples were packaged at the PHCs/subcentres using the triple-layer packaging method to ensure safe transport, as per ICMR guidelines for drone transport of biological samples.^14^

### Statistical analysis

Data were analysed using Jamovi 2.6.44. Normality was checked using the Shapiro–Wilk test, and appropriate statistical tests were applied accordingly. Descriptive statistics included mean ± standard deviation (SD) and median with interquartile range (IQR). The Mann–Whitney *U* test was applied to compare outcomes between phases (*P* < 0.05). Given the operational design and limited sample size in the pre-drone phase, multivariate regression was not conducted.

### Ethical statement

Administrative approvals were obtained from the District Collector, State TB Officer, District Medical and Health Officer, and District TB Officer. Approvals for drone operations and airspace usage were granted by the Directorate General of Civil Aviation, Government of India, and the Rachakonda Police Commissionerate, Government of Telangana (Ref. No. M4/Events/RCK/305/2024).^15^ The study received ethics approval from the Institutional Ethics Committee of AIIMS Bibinagar (IEC No: AIIMS/BBN/IEC/JULY/2022/164). Written informed consent was obtained at site from all adult participants, and for minors (12–17 years), written parental consent and participant assent were obtained prior to enrolment.

## RESULTS

A total of 840 participants were included, comprising 206 in the pre-drone phase and 634 in the drone phase. Most participants resided in remote villages located 10–30 km from the nearest TUs, in areas characterised by hilly terrain, poor road connectivity, and limited public transport. The mean age (SD) of participants was 49.1 (16.1) years in the pre-drone group and 59.8 (15.6) years in the drone group. The majority of the participants were male in both phases, with 62.6% in pre-drone and 60.2% in drone group. More than 80% of participants in both phases belonged to households below the poverty line and had no formal education. Monthly income was low across both groups, with 71.4% of pre-drone participants and 91.3% of drone-phase participants reporting earnings below INR 5,000 (≈USD 60) per month. A cough lasting more than 2 weeks was the most common presenting symptom observed: 96.6% in the pre-drone-phase participants and 73.2% in the drone-phase participants (Table 2).

**Table 2.** Distribution of study participants based on socio-demographic characteristics, clinical features, and co-morbidities across pre-drone and drone phases.

Variable	Pre-drone phase (N = 206)	During-drone phase (N = 634)
	N (%)	N (%)
Mean age in years (± SD)	49.14 ± 16.1	59.8 ± 15.6
Age groups in years		
≤20	11 (5.34)	15 (2.37)
21–40	49 (23.79)	64 (10.09)
41–60	95 (46.12)	214 (33.75)
61–80	47 (22.82)	320 (50.47)
≥81	3 (1.46)	21 (3.31)
Gender distribution		
Men	129 (62.62)	382 (60.25)
Women	77 (37.38)	252 (39.75)
Marital status		
Married	189 (91.75)	610 (96.21)
Unmarried	14 (6.80)	21 (3.31)
Divorced/widowed/separated	3 (1.46)	3 (0.47)
Education level		
Illiterate	97 (47.09)	510 (80.44)
Primary school	39 (18.93)	43 (6.78)
Middle school	9 (4.37)	29 (4.57)
High school	10 (4.85)	27 (4.26)
Higher secondary school	35 (16.99)	18 (2.84)
Diploma/graduation/PG	16 (7.77)	7 (1.10)
Poverty status		
APL	30 (14.56)	12 (1.89)
BPL	175 (84.95)	554 (87.38)
Monthly income		
0 ($0)^**A**^	57 (27.67)	60 (9.46)
≤5,000 (≤$60)	90 (43.69)	519 (81.86)
5,001–10,000 ($60–$120)	26 (12.62)	24 (3.79)
10,001–15,000 ($120–$180)	11 (5.34)	25 (3.94)
15,001–20,000 ($180–$240)	19 (9.22)	4 (0.63)
>20,001 ($240)	3 (1.46)	2 (0.32)
Clinical features		
Symptoms		
Cough	199 (96.60)	464 (73.19)
Fever	162 (78.64)	117 (18.45)
Weight loss	116 (56.31)	43 (6.78)
Shortness of breath	89 (43.20)	126 (19.87)
Blood in sputum	10 (4.85)	2 (0.32)
Co-morbidities		
Hypertension	42 (20.39)	141 (22.24)
Diabetes	29 (14.08)	93 (14.67)
COPD/Asthma	6 (2.92)	25 (3.94)
Others^**B**^	18 (8.74)	5 (0.79)

SD = standard deviation; PG = post-graduation; APL = above poverty line; BPL = below poverty line; COPD = chronic obstructive pulmonary disease.

A Students, housewives.

B HIV, thyroid dysfunction, chronic kidney disease, heart disease, stroke.

### Impact on turnaround time (TAT) and delays

The median TAT for diagnosis decreased from 15 days (IQR: 10–20) in the pre-drone phase to 5 days (IQR: 2–9) in the drone phase, with the mean (SD) TAT reducing from 16.6 days (18.1) to 6.9 days (8.3) (*P* < 0.001). Patient delay had a median of 6 days (IQR: 4–10) in the pre-drone phase and 6 days (IQR: 3–13) in the drone phase. Although the median remained unchanged, the mean (SD) patient delays decreased from 16.6 days (45.8) in the pre-drone phase to 9.2 days (10.1) in the drone phase, reflecting a reduction in extreme delays, and diagnostic delay decreased from a median of 15 days (IQR: 10–20) to 6 days (IQR: 2–10), with the mean (SD) diagnostic delay reducing from 17.6 days (18.0) to 7.9 days (8.5) (*P* < 0.001) (see Table 3).

**Table 3.** Descriptive and inferential analysis of delays in the TB diagnostic pathway across study phases.

Variable	Phase	Mean ± SD	Median (IQR)	U^**A**^	*P* value
TAT (days)	Pre-drone	16.60 ± 18.10	15.0 (10–20)	24,645	<0.001^**B**^
	Drone	6.96 ± 8.34	5.00 (2–9)	24,645	<0.001^**B**^
Patient delay (days)	Pre-drone	16.60 ± 45.80	6.00 (4–10)	50,363	0.067
	Drone	9.15 ± 10.10	6.00 (3–13)	50,363	0.067
Diagnostic delay (days)	Pre-drone	17.60 ± 18.00	15.0 (10–20)	24,115	<0.001^**B**^
	Drone	7.88 ± 8.51	5.00 (2–10)	24,115	<0.001^**B**^

χ^2^ test of independence: χ^2^ (2, N = 840) = 408.03.

IQR = interquartile range; SD = standard deviation; TAT = turnaround time.

A The U value is the Mann–Whitney *U* test statistic.

B All *P* values based on the Mann–Whitney *U* test due to non-normal distribution (Shapiro–Wilk test).

### Impact on out-of-pocket expenditure (OOPE)

Drone-enabled transport was associated with a substantial decline in patient expenditure. In the pre-drone phase, the mean OOPE per patient was INR 9,451 (USD 113.4) with a median of INR 2,735 (USD 32.8); meanwhile, during the drone phase, the mean OOPE fell to INR 90.9 (USD 1.1) and the median to INR 0, indicating that most patients incurred no travel or diagnostic costs (Table 4).

**Table 4.** Out-of-pocket expenditure (OOPE) per patient in pre- and post-drone phases.

Cost component	Phase	Mean cost (SD) in INR	Mean cost (SD) in USD	*P* value
Direct medical cost	Pre-Drone	7,017 (20,834)	84.20 (250.01)	<0.001
	Drone	75.8 (693)	0.91 (8.32)	
Direct nonmedical cost	Pre-Drone	787 (2,248)	9.44 (26.98)	<0.001
	Drone	12.4 (202)	0.15 (2.42)	
Indirect cost	Pre-Drone	1,646 (7,611)	19.75 (91.33)	<0.001
	Drone	2.71 (34.3)	0.03 (0.41)	
Total OOPE	Pre-Drone	9,451 (23,915)	113.41 (286.98)	<0.001
	Drone	90.9 (745)	1.09 (8.94)	

### Impact on reporting delays

The proportion of patients receiving same-day results increased slightly from 6.3% in the pre-drone phase to 7.4% during the drone phase, while next-day results rose markedly from 1.5% to 76.3%. Conversely, the proportion experiencing delays more than 2 days fell from 92.2% in the pre-drone phase to 16.3% in the drone phase (Table 5).

**Table 5.** Association between study phase and the time taken for reporting of TB diagnosis.

Indicator	Same-day result reporting	Next-day result reporting	Greater than 2 days result reporting	*P* value
Pre-drone phase (N = 206), N (%)	13 (6.3)	3 (1.5)	190 (92.2)	<0.001
Drone phase (N = 634), N (%)	47 (7.4)	484 (76.3)	103 (16.3)	<0.001

## DISCUSSION

This study evaluated the impact of integrating drone-based sputum transport into the TB diagnostic network in the rural Yadadri-Bhuvanagiri district of Telangana, India. To our knowledge, this is the first programme-based study conducted in collaboration with the NTEP of India that assessed drone transport over an extended duration. The intervention was associated with significant reductions in TAT, diagnostic delays, patient delays, and OOPE, highlighting potential of drone logistics to enhance equitable access to TB care in India. During the drone phase, participants were generally older, less educated, and economically disadvantaged, and the sample size was substantially larger than in the pre-drone phase, and this imbalance likely reflects the operational expansion of the intervention, and while it suggests improved diagnostic reach among underserved communities, it also needs to be considered when interpreting comparative outcomes. These findings are consistent with earlier studies, which suggested that drone-enabled diagnostics can contribute to inclusive and equitable TB care.^3,16^ The observed reductions in TAT and variability in patient delay reflect improved reliability and coordination in diagnostic service delivery. Similar improvements in sample transport efficiency have been reported by Gairolla et al.^17^ in Uttarakhand, India, where drones reduced delays in hilly terrain.

A 99% reduction in OOPE in our study further underscores the economic benefit of drone-enabled sputum transport. In the pre-drone phase, high costs were incurred due to long travel to TUs, augmented by wage loss and stigma associated with travelling while symptomatic. In the drone phase, sputum was collected directly at nearby PHCs or subcentres located within patients’ villages, which could generally be accessed by walking or at a negligible cost of INR 50 (≈USD 0.60) or less. This explains the median OOPE of zero during the drone phase. These results support findings by Pradipta et al.^3^ who highlighted financial vulnerability as a major barrier to TB care, and by Thakur et al.^16^ and Lakshman et al.^8^ who noted drones as a cheaper alternative for sputum transport.

Our results also align with global evidence that drones can overcome geographic and infrastructural barriers to health care. Poljak and Šterbenc^18^ as well as van Veelen et al.^19^ demonstrated that drones can traverse terrain-related barriers to improve access to essential services. The operational success observed in this study also mirrors findings from Aggarwal et al.^20^ in Northeast India, where drones successfully delivered over 20,000 medical units across Manipur and Nagaland despite severe logistical challenges.

However, a few limitations should also be noted. First, the demographic differences between phases may have introduced confounding effects. Second, the pre-post study does not include a concurrent control group limiting the ability to establish causality. Third, the patient expenditure data were self-reported and therefore may have been affected by recall bias. Lastly, due to operational issues and relatively short duration of intervention, assessment of longer-term outcomes such as treatment initiation and adherence was not possible, and further research is required.

## CONCLUSION

This study provides evidence that drone operations resulted in a significant reduction in TAT and delays at various levels for TB diagnosis under NTEP in the hard-to-reach areas of the Yadadri-Bhuvanagiri district of Telangana. The intervention showed reductions in TAT, delays, and OOPE, benefiting the vulnerable population. This study can help highlight the potential of integrating innovative strategies like these to strengthen TB care delivery as well as inch closer to the elimination of TB.
